# Longitudinal and cross-sectional investigations of long-term potentiation-like cortical plasticity in bipolar disorder type II and healthy individuals

**DOI:** 10.1038/s41398-018-0151-5

**Published:** 2018-05-24

**Authors:** Nathalia Zak, Torgeir Moberget, Erlend Bøen, Birgitte Boye, Trine R. Waage, Espen Dietrichs, Nina Harkestad, Ulrik F. Malt, Lars T. Westlye, Ole A. Andreassen, Stein Andersson, Torbjørn Elvsåshagen

**Affiliations:** 1 Norwegian Centre for Mental Disorders Research (NORMENT), KG Jebsen Centre for Psychosis Research, Oslo University Hospital, Oslo, Norway; 2 Institute of Clinical Medicine, University of Oslo, Oslo, Norway; 3 Department of Psychiatry, Diakonhjemmet Hospital, Oslo, Norway; 4 Section of Psychosocial Oncology, Division of Cancer Medicine, Oslo University Hospital, Oslo, Norway; 5 Department of Behavioural Sciences in Medicine, University of Oslo, Oslo, Norway; 6 Department of Psychology, University of Oslo, Oslo, Norway; 7 Department of Neurology, Oslo University Hospital, Oslo, Norway; 8 Department of Biological and Medical Pscyhology, University of Bergen, Bergen, Norway; 9 Department of Research and Education, Oslo University Hospital, Oslo, Norway

## Abstract

Visual evoked potential (VEP) plasticity is a promising assay for noninvasive examination of long-term potentiation (LTP)-like synaptic processes in the cerebral cortex. We conducted longitudinal and cross-sectional investigations of VEP plasticity in controls and individuals with bipolar disorder (BD) type II. VEP plasticity was assessed at baseline, as described previously (Elvsåshagen et al. Biol Psychiatry 2012), and 2.2 years later, at follow-up. The longitudinal sample with VEP data from both time points comprised 29 controls and 16 patients. VEP data were available from 13 additional patients at follow-up (total *n* = 58). VEPs were evoked by checkerboard reversals in two premodulation blocks before and six blocks after a plasticity-inducing block of prolonged (10 min) visual stimulation. VEP plasticity was computed by subtracting premodulation VEP amplitudes from postmodulation amplitudes. Saliva samples for cortisol analysis were collected immediately after awakening in the morning, 30 min later, and at 12:30 PM, at follow-up. We found reduced VEP plasticity in BD type II, that impaired plasticity was present in the euthymic phases of the illness, and that VEP plasticity correlated negatively with depression severity. There was a positive association between VEP plasticity and saliva cortisol in controls, possibly reflecting an inverted U-shaped relationship between cortisol and synaptic plasticity. VEP plasticity exhibited moderate temporal stability over a period of 2.2 years. The present study provides additional evidence for impaired LTP-like cortical plasticity in BD type II. VEP plasticity is an accessible method, which may help elucidate the pathophysiological and clinical significance of synaptic dysfunction in psychiatric disorders.

## Introduction

Bipolar disorder (BD) type I and II affect 2–3 % of the population and can lead to marked impairments in social and occupational functioning^1–3^. The estimated heritability of BD is ~ 0.7^4,5^, yet its precise pathophysiological basis remains unknown. Consequently, current therapeutic options may not target fundamental illness processes and remain insufficient for a substantial number of patients^6,7^. The clarification of central pathophysiological mechanisms is therefore a critical step toward improved outcomes in BD.

Synaptic dysfunction is one of the leading candidate mechanisms across psychiatric illnesses^8–13^. In particular, preclinical studies and genetic investigations have implicated synaptic plasticity in the etiology and treatment of BD, major depressive disorder (MDD), autism spectrum disorder, and schizophrenia^13–23^. Despite these findings, there is a paucity of clinical evidence supporting synaptic dysfunction in psychiatric disorders, mainly due to a lack of methods for noninvasive measurements of synaptic function and plasticity in humans. However, electroencephalography (EEG)-based measurement of visual cortex plasticity has in recent years emerged as a promising assay for *in vivo* assessment of synaptic function and plasticity^24,25^. Previous studies showed that repeated visual stimulation-induced increases of the human visual evoked potential (VEP), i.e., an EEG signal that primarily reflects postsynaptic potentials in the visual cortex^26–30^. Further investigations found that VEP plasticity was reduced in BD type II, MDD, and schizophrenia^28–30^. Although the precise neural substrates for VEP plasticity in humans remain to be clarified, detailed studies in rodents showed that VEP increases induced by repetitive visual stimulation is long-lasting, stimulus-specific, and depends on synaptic N-methyl-d-aspartate (NMDA) and alpha-amino-3-hydroxy-5-methyl-4-isoxazolepropionic acid receptors and protein kinase Mζ^31–33^. These are all core features of long-term potentiation (LTP), which is the best characterized form of synaptic plasticity^34–36^. Together, these results indicate that VEP plasticity is reduced and may reflect impairments of cortical LTP-like synaptic processes, in BD type II, MDD, and schizophrenia.

Despite these promising findings, more research is needed to clarify the mechanisms, translational potential, and clinical utility of VEP plasticity in BD and other psychiatric illnesses. Impaired VEP plasticity across psychiatric illnesses^28–30^ may suggest that nonspecific mechanisms such as stress- and cortisol-related synaptic dysfunction^37,38^ could underlie the plasticity reductions. Moreover, the relationships between VEP plasticity and clinical characteristics of psychiatric illnesses remain to be clarified. In addition, there are to our knowledge no longitudinal studies of VEP plasticity and its temporal stability in healthy volunteers and patient groups remains unknown.

We previously found plasticity of the VEP in healthy controls and reduced plasticity in BD type II^29^. Here, we conducted longitudinal and cross-sectional investigations of VEP plasticity in individuals with BD type II and controls with the following main aims: (1) to test the reproducibility of impaired VEP plasticity in BD type II and to assess the relationship between mood state and plasticity, (2) to examine the relationship between saliva cortisol and VEP plasticity in controls and BD type II, and (3) to examine the temporal stability of VEP plasticity.

## Methods and materials

### Participants and clinical examinations

We assessed VEP plasticity at Oslo University Hospital in 40 controls and 26 individuals with BD type II at baseline, as described previously^29^. At follow-up, on average 2.2 years later, 33 of the controls and 18 of the patients again underwent the VEP plasticity examinations. Two patients and four controls were excluded from the analyses owing to technical issues during EEG recording and insufficient data quality, thus the longitudinal sample comprised 29 controls and 16 patients. Moreover, 16 additional patients and one new control were included at follow-up to further assess the relationship between VEP plasticity and saliva cortisol and mood state. Owing to technical issues and insufficient data quality, three of the new patients and the new control were excluded from the analyses. Thus, the cross-sectional patient sample at follow-up included 29 participants. In controls, the longitudinal sample and the cross-sectional sample at follow-up were identical and comprised 29 individuals.

The patients were recruited from psychiatric outpatient clinics in the Oslo area. Clinical examinations at baseline and follow-up were carried out by senior psychiatrists (i.e., authors EB, BB, and UFM) at a university department specializing in the evaluation and treatment of mood disorders. Axis I diagnoses and psychiatric comorbidities were determined with the Mini-International Neuropsychiatric Interview, DSM-IV criteria version 5.0^39^. Alcohol and drug use were assessed with the Alcohol Use Scale and the Drug Use Scale^40^, respectively. Mood state was assessed at the day of EEG recording for the large majority of participants and within 3 days of the recording for all participants. Assessments were carried out by trained physicians (i.e., authors EB, BB, UFM, and TE) using the Montgomery–Asberg Depression Rating Scale (MADRS)^41^ and the Young Mania Rating Scale (YMRS)^42^. These physicians underwent a day course of MADRS and YMRS prior to the present study, which included estimation of their intraclass correlation coefficient_3.1_ (*ICC*_3.1_); all *ICC*_3.1_’s were > 0.8.

Controls with no previous or current psychiatric illness were recruited through local advertising and underwent a full examination similar to that of the patients at baseline and follow-up. The exclusion criteria for all subjects were: age below 18 or above 50 years, history of neurological or other severe chronic somatic disorder, and pregnancy. One patient had experienced a mild head injury with loss of consciousness for > 1 min. However, the patient did not have any clinical or magnetic resonance imaging-detectable cerebral sequela and was included in the study. Otherwise, no participant reported head injury with loss of consciousness for > 1 min. All subjects had normal or corrected-to-normal visual acuity. The Regional Ethical Committee of South-Eastern Norway approved the study, and all subjects provided written informed consent to participate.

### Experimental paradigm

**Fig. 1:**
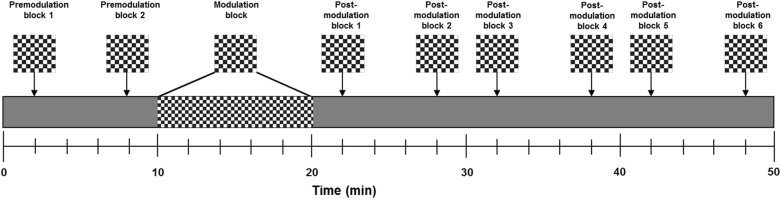
Experimental setup. The experimental paradigm described by Normann et al.^28^ was used at baseline, as described previously, and at follow-up. VEPs were evoked by checkerboard reversals (check size = 0.5°; 2 reversals/sec) in two premodulation blocks before and six blocks after a plasticity-inducing modulation block consisting of 10 min of checkerboard reversal stimulation (check size = 0.5°; 2 reversals/sec). The premodulation blocks were initiated 2 and 8 min after the start of the experiment, and the modulation block was initiated 2 min after the last premodulation block. The postmodulation blocks were performed 2, 8, 12, 18, 22, and 28 min after the end of the modulation block. A gray screen was displayed in the intervals between checkerboard stimulation. Participants were instructed to focus on a filled red circle (0.1°) in the center of the screen during the experiment and were allowed to listen to music. The participants were monitored throughout the experiment to ensure that they followed instructions and maintained attention. VEP, visual evoked potential

The experimental paradigm described by Normann et al.^28^. was used at baseline^29^ and follow-up. VEPs were evoked by checkerboard reversals (check size = 0.5°; 2 reversals/sec) in two premodulation blocks before and six blocks after a plasticity-inducing modulation block (Fig. 1). In each pre- and postmodulation block, 40 checkerboard reversals were presented within 20 s. In the modulation block, VEPs were evoked by checkerboard reversals (check size = 0.5°; 2 reversals/sec) for 10 min. The premodulation blocks were initiated 2 and 8 min after the start of the experiment, and the modulation block was initiated 2 min after the last premodulation block. Then, the postmodulation blocks were performed 2, 8, 12, 18, 22, and 28 min after the end of the modulation block. A gray screen was displayed between checkerboard stimulation. Participants focused on a filled red circle (0.1°) in the center of the screen during the experiment. They were monitored throughout the experiment to ensure that they followed instructions and maintained attention, and were allowed to listen to music. The visual stimuli were presented with E-Prime 1.1 (Psychology Software Tools, Sharpsburg, Pennsylvania) on a Samsung Syncmaster 2493HM LCD screen (Samsung Electronics Nordic AB, Oslo, Norway). To ensure high timing accuracy, a photodiode from the Black Box Toolkit® (Sheffield, UK) was used and VEP latencies were corrected accordingly.

### Recording and analysis of the VEP

VEP plasticity was assessed using EEG data from the Oz electrode at both time points. The baseline examination also included mismatch negativity and oddball paradigms. Continuous EEG activity was therefore recorded from 15 monopolar silver/silver chloride electrodes for analyses of these paradigms at baseline. However, the follow-up examination only comprised the VEP plasticity paradigm and only three electrodes were therefore used (O1, Oz, and O2). All impedances were maintained below 5 kΩ and the ground and reference electrodes were attached to the forehead (AFz). Eye movements were recorded with bipolar electrodes placed at the sub- and supraorbital regions and at the lateral canthi of each eye. EEG activity was recorded at 250 Hz with an amplifier band-pass of 0.05–100 Hz. Offline EEG analysis was conducted with EEGLAB^43^, run on MATLAB 7.6.0. (MathWorks, Natick, Massachusetts). The EEG was first high-pass filtered at 1 Hz, and segmented into epochs starting 150 msec before and continuing 350 msec after the onset of each checkerboard reversal. All epochs containing eye movement-related activity were removed from analyses.

Epochs were then shortened (−50 to 350 msec) and baseline-corrected (−50 to 0 msec), and epochs with amplitudes exceeding ± 100 µV on any of the occipital channels (O1, Oz, O2) were rejected. The epoched EEG was finally low-pass filtered at 30 Hz and averaged to block-specific VEPs. Peak amplitudes and latencies for the C1, P1, N1, and the P1–N1 peak-to-peak amplitudes were obtained from the Oz electrode at the occipital head; amplitudes were measured relative to the 50 msec baseline.

### Saliva collection and cortisol analysis

Saliva samples for cortisol analysis were collected using Salivette® Cortisol swabs (Sarstedt AG & Co, Nümbrecht, Germany) and analyzed with a Cortisol Saliva Luminescence Immunoassay (IBL International, Hamburg, Germany) according to the manufacturers´ instructions. Saliva samples were obtained the day after the VEP experiment at three times: immediately after awakening in the morning, 30 min after the first collection, and at 12:30 PM. Participants were instructed to not brush teeth and to refrain from physical activity, nicotine, and caffeine before saliva collection and to not eat or drink the last 30 min before the samples were obtained. Saliva cortisol was averaged across the three collections. We also computed the cortisol awakening response (saliva cortisol 30 min post awakening minus cortisol at awakening), as a previous study found that the cortisol awakening response was associated with transcranial magnetic stimulation (TMS)-induced motor cortex plasticity^44^. Complete cortisol data were missing for one control and two individuals with BD type II.

### Statistical analyses

All statistical analyses were conducted with SPSS version 24 for Windows (IBM Corp., Armonk, NY) and a two-tailed *p* value of < .05 was considered significant. VEP amplitudes from the two premodulation and from the six postmodulation recordings were averaged as premodulation and postmodulation VEP, respectively. To examine the effect of the modulation block on the VEP, the C1, P1, N1, and P1–N1 premodulation amplitudes were compared with the corresponding postmodulation amplitudes with repeated measures analysis of variance (ANOVA) in controls and patients separately. VEP plasticity was computed by subtracting premodulation VEP amplitudes from the corresponding postmodulation amplitudes.

Saliva cortisol was compared between groups using ANOVA. VEP plasticity scores were subjected to ANOVAs and analyses of covariance, after testing the assumption of homogeneity of regression slopes, to examine the effects of group, covarying for saliva cortisol, premodulation VEP amplitudes, and educational level (in the case of significant group differences for the latter variables) and the effect of medication (controls vs. unmedicated patients vs. medicated patients, Bonferroni corrected for the three contrasts). The relationships between VEP plasticity and mood state, saliva cortisol, and other clinical variables were assessed with Pearson correlation, Spearman´s rank correlation, and repeated measures ANOVA. The temporal stability of VEP plasticity was examined using Pearson correlation and *ICC*_3.1_.

## Results

### Demographic and clinical variables

Demographic and clinical variables for the longitudinal and the cross-sectional samples are shown in Table 1. There were no significant group differences in age or gender. In the longitudinal sample, controls had a higher educational level than patients; otherwise there were no significant group differences. Eighteen out of 29 patients were euthymic (MADRS score < 11 and YMRS score < 8), eight patients were depressed (MADRS score 12–23) and three patients were hypomanic (YMRS score 8) at follow-up examinations.

**Table 1:** Demographic and clinical characteristics of the longitudinal and the cross-sectional samples of patients with bipolar disorder type II and healthy controls

	Longitudinal sample^a^			Cross-sectional sample at follow-up		
	BD type II (*n* = 16)	Control group (*n* = 29)	*p*	BD type II (*n* = 29)	Control group (*n* = 29)	*p*
Age, years, mean (SD)	32.7 ± 7.5	33.1 ± 9.4	0.87	35.5 ± 7.9	35.6 ± 9.6	0.99
Female, *n* (%)	9 (56)	16 (55)	0.94	19 (66)	16 (55)	0.42
Education level, *n* (%)						
0–10 years	2 (12.5)	0 (0)		2 (7)	0 (0)	
11–13 years	4 (25)	3 (10)		8 (28)	2 (7)	
14–17 years	8 (50)	11 (38)		9 (31)	11 (38)	
17 + years	2 (12.5)	15 (52)	0.02	10 (34)	16 (55)	0.07
MADRS, mean (SD)	11.9 ± 6.5	1.3 ± 2.2	< 0.001	8.8 ± 6.8	1.3 ± 1.6	< 0.001
YMRS, mean (SD)	3.0 ± 3.4	0.3 ± .8	< 0.001	2.4 ± 2.5	0.3 ± .7	< 0.001
Euthymia (MADRS < 11, YMRS < 8)	10			18		
Depression (MADRS > 11)	5			8		
Hypomania (YMRS ≥ 8)	1			3		
Medication, *n* (%)						
Unmedicated	6 (38)			7 (24)		
Antidepressants	6^b^ (38)			7^c^ (24)		
Lamotrigine	6 (38)			16 (55)		
Lithium	1 (6)			0 (0)		
Quetiapine	1 (6)			3 (10)		
Methylphenidate	0 (0)	n.a.		2 (7)	n.a.	
Duration of illness, years, mean (SD)	17.3 ± 8.1	n.a.		18.0 ± 7.3	n.a.	
Social phobia, *n* (%)	4 (25)	0 (0)		10 (34)	0 (0)	
Panic disorder, *n* (%)	6 (38)	0 (0)		18 (62)	0 (0)	
General anxiety disorder, *n* (%)	2 (13)	0 (0)		2 (7)	0 (0)	

^a^Characteristics at baseline

^b^Antidepressants were escitalopram, citalopram, bupropion, mirtazapine, and fluoxetine

^c^Antidepressants were escitalopram, citalopram, bupropion, mirtazapine, venlafaxine, sertraline, and mianserin

### **VEPplasticity of the longitudinal sample**

#### **VEPplasticity of the longitudinal sample at baseline**

We previously reported significant VEP plasticity in healthy controls and impaired plasticity of the P1–N1 amplitude in individuals with BD type II at baseline (when the whole sample of 40 controls and 26 individuals with BD type II was analyzed)^29^. Here, we reran the VEP analyses for the longitudinal sample at baseline (*n* = 45) and found no significant group differences in the C1, P1, N1, or the P1–N1 amplitudes of the premodulation blocks (all *p* > 0.05). There was significant plasticity of the P1 (*F*_1,28_ = 8.36, *p* = 0.007), N1 (*F*_1,28_ = 4.88, *p* = 0.036), and P1–N1 (*F*_1,28_ = 34.95, *p* < 0.001) amplitudes in controls (Supplementary Figure 1A), but not in patients (Supplementary Figure 1B; all *p* > 0.05). Relative to controls, there was significantly reduced P1–N1 plasticity (*F*_1,43_ = 13.82, *p* = 0.001) and a trend towards reduced P1 plasticity that did not reach significance (*p* = 0.065) in patients (Supplementary Figure 1C), consistent with the previously published results for the whole sample (*n* = 66)^29^. There was a significant negative correlation of P1 and N1 amplitudes in controls (*r* = −0.64, *p* < 0.001) and in patients (*r* = −0.77, *p* < 0.001).

#### VEP plasticity of the longitudinal sample at follow-up

**Fig. 2:**
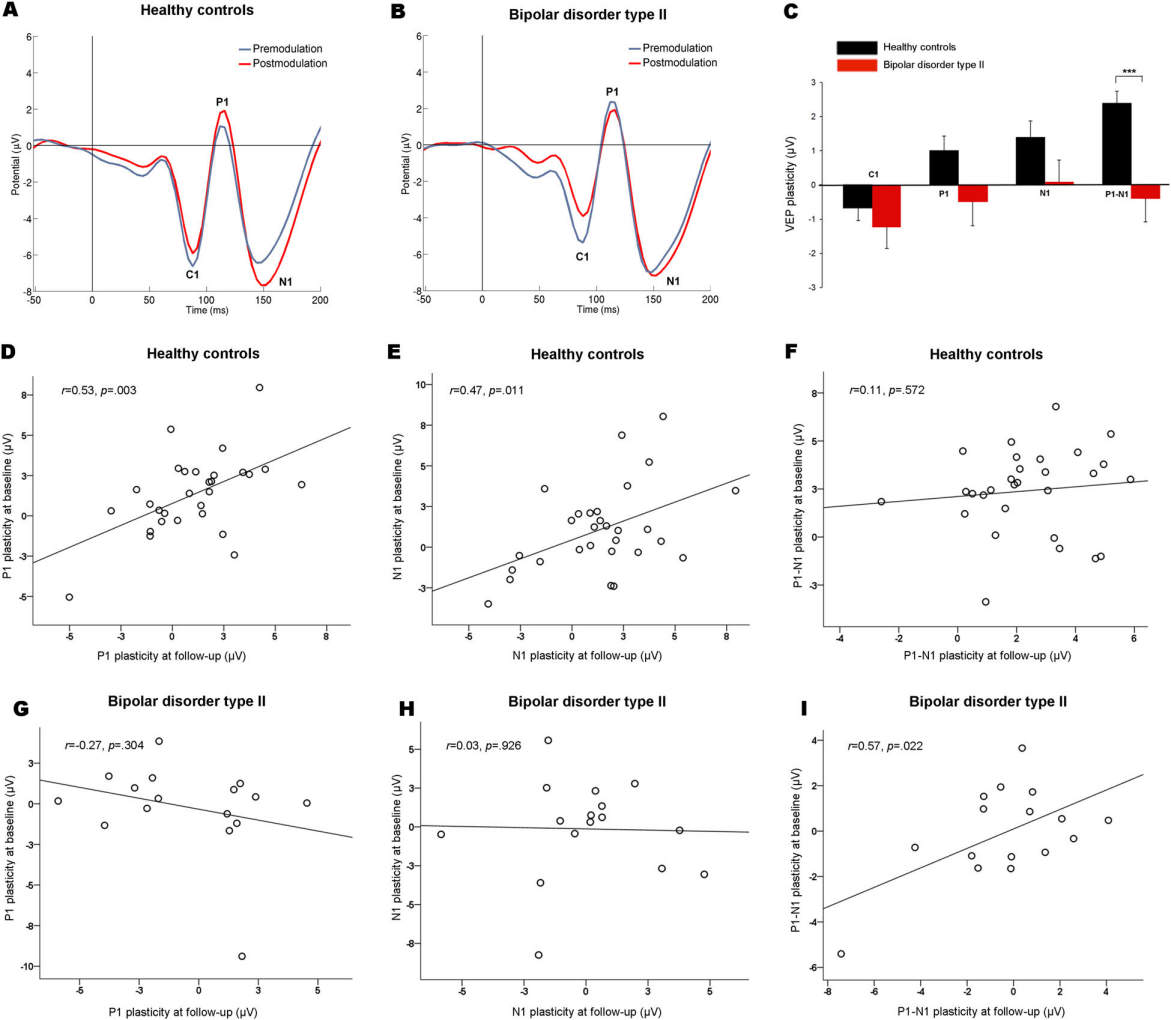
VEP plasticity of the longitudinal sample at follow-up. **a** Grand average premodulation (blue) and postmodulation (red) VEP in controls (*n* = 29). The modulation block resulted in significant plasticity of the P1, N1, and P1–N1 amplitudes. **b** Grand average premodulation (blue) and postmodulation (red) VEP in patients with BD type II (*n* = 16). There was no significant P1, N1, or P1–N1 plasticity in the patient group. **c** P1–N1 plasticity was significantly reduced in patients with BD type II relative to controls. There was no significant group difference in C1 or N1 plasticity; however, there was a trend toward reduced P1 plasticity in patients (*p* = 0.07). The group difference in P1–N1 plasticity remained significant after controlling for cortisol, premodulation amplitude, and educational level. ***p < 0.001. Error bars represent the s.e.m. **d** Temporal stability of VEP plasticity in controls and patients with BD type II. There was a significant positive correlation between baseline and follow-up P1 plasticity and **e** N1 plasticity, but not **f** P1–N1 plasticity in controls. **g** There was no significant correlation between baseline and follow-up P1 plasticity or **h** N1 plasticity, however, **i** there was a significant correlation for baseline and follow-up P1–N1 plasticity in patients with BD type II.VEP, visual evoked potential. BD, bipolar disorder

At follow-up, checkerboard reversal stimulation produced the expected VEP amplitudes at the premodulation blocks, with C1 at 88.1 ± 0.9 msec (mean ± s.e.m), P1 at 114.3 ± 0.8 msec, and N1 at 147.1 ± 1.9 msec in controls (Fig. 2a) and with C1 at 87.4 ± 1.6 msec, P1 at 114.3 ± 1.3 msec, and N1 at 151.0 ± 3.1 msec in patients (Fig. 2b). There were no differences between patients and controls in the latencies of the premodulation or postmodulation amplitude peaks (all *p* values > 0.05). P1 and N1 latencies of the postmodulation blocks were significantly increased relative to the premodulation latencies in controls (115.8 ± 0.8 msec vs. 114.3 ± 0.8 msec, *F*_1,28_ = 11.17; *p* = 0.002 and 150.5 ± 1.8 msec vs. 147.1 ± 1.9 msec, *F*_1,28_ = 32.31; *p* < 0.001, respectively). There were no significant latency changes in patients and no significant effect of group on changes in latencies from premodulation to postmodulation blocks (all *p* values > 0.05). Patients had significantly greater P1–N1 amplitude at the premodulation blocks than controls (*F*_1,43_ = 4.51; *p* = 0.04), whereas no significant group differences were found for the C1, P1, or the N1 premodulation amplitudes (all *p* > 0.05). There was significant plasticity of the P1 (*F*_1,28_ = 5.31, *p* = 0.03), N1 (*F*_1,28_ = 7.89, *p* = 0.009), and P1–N1 (*F*_1,28_ = 157.48, *p* < 0.001) amplitudes in controls, but not in patients (all *p* > 0.05). In controls, the P1–N1 plasticity effect was significant for all postmodulation blocks (postmodulation block 1: *F*_1,28_ = 124,25, *p* < 0.001, block 2: *F*_1,28_ = 16.13, *p* < 0.001, block 3: *F*_1,28_ = 13.25, *p* = 0.001, block 4: *F*_1,28_ = 5.10, *p* = 0.032, block 5: *F*_1,28_ = 15.43, *p* = 0.001, and block 6: *F*_1,28_ = 15.42, *p* = 0.001; thus five out of six blocks surviving Bonferroni correction, *p* < 0.05/6) (Figure S2A). There was a significant negative correlation between modulation of P1 and N1 amplitudes in controls (*r* = −0.72, *p* < 0.001) and in patients (*r* = −0.50, *p* = 0.047). There was significantly reduced P1–N1 plasticity (*F*_1,43_ = 16.26; *p* < 0.001) and a trend towards reduced P1 plasticity (*F*_1,43_ = 3.48; *p* = 0.07), in patients relative to controls (Fig. 2c). There was no significant group-salivary cortisol level interaction, group-premodulation amplitude interaction or group-educational level interaction for P1–N1 plasticity (all *p’s* > 0.1), and the group difference remained significant after adjusting for these covariates (*F*_1,34_ = 8.51, *p* = 0.006).

#### Temporal stability of VEP plasticity in controls and patients

In controls, there was a significant association between P1 plasticity at baseline and follow-up (*r* = 0.53, *p* = 0.003; *ICC*_3.1_ = 0.53; Fig. 2d). There was also a significant relationship between N1 plasticity at baseline and follow-up (*r* = 0.57, *p* = 0.011; *ICC*_3.1_ = 0.47; Fig. 2e), whereas no significant association was found for P1–N1 plasticity (*r* = 0.11, *p* = 0.57; *ICC*_3.1_ = 0.11; Fig. 2f), in controls. In patients, there were no significant associations for P1 or N1 plasticity (*r* = −0.27, *p* = 0.30; *ICC*_3.1_ = −0.27 and *r* = −0.03, *p* = 0.093; *ICC*_3.1_ = 0.03, respectively, Fig. 2g, h), yet a significant association for P1–N1 plasticity was found (*r* = 0.57, *p* = 0.022; *ICC*_3.1_ = 0.55; Fig. 2i), when the baseline and follow-up VEP plasticity results were compared.

#### Explorative longitudinal analyses

There was no significant effect of time and no significant group**×**time interaction effect on P1, N1, or P1–N1 plasticity (all *p* > 0.05). There was no significant effect of adding a psychotropic drug on VEP plasticity changes from baseline to follow-up (patients unmedicated at baseline and medicated at follow-up (*n* = 5) vs. other patients (*n* = 11) vs. controls (*n* = 29); all *p* > 0.05; see also Table S5 for details). There were no significant associations between P1, N1, or P1–N1 plasticity at baseline and number of depressive and hypomanic episodes between baseline and follow-up (all *p* > 0.05).

### **VEPplasticityandsaliva cortisol of the cross-sectional sample at follow-up**

#### Saliva cortisol

**Fig. 3:**
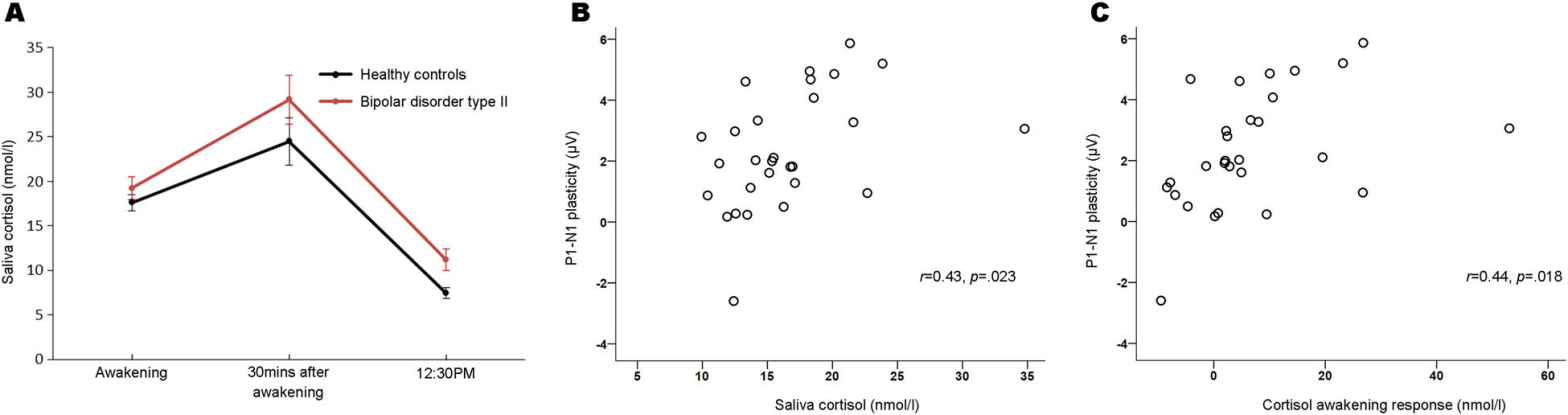
Saliva cortisol and VEP plasticity. **a** Saliva cortisol was collected the day after the VEP experiment at three times: immediately after awakening in the morning, 30 min after the first collection, and at 12:30 PM. Saliva cortisol was averaged across the three collections and was significantly increased in patients with BD type II relative to controls. **b** In controls, there was a significant positive correlation between saliva cortisol and plasticity of the P1–N1 amplitude, indicating greater plasticity with higher cortisol levels. **c** There was also a significant positive correlation between the cortisol awakening response and plasticity of the P1–N1 amplitude in controls. VEP, visual evoked potential. BD, bipolar disorder

The saliva cortisol analyses showed the expected morning awakening response in patients and controls at follow-up (Fig. 3a); there was no significant group difference in the awakening response (*p* = 0.37). Saliva cortisol averaged across the three collections was higher in patients than controls (*F*_1,53_ = 5.35, *p* = 0.025) and saliva cortisol at 12:30 PM was significantly increased in patients (*F*_1,55_ = 6.59, *p* = 0.013).

#### VEP plasticity and clinical variables

**Fig. 4:**
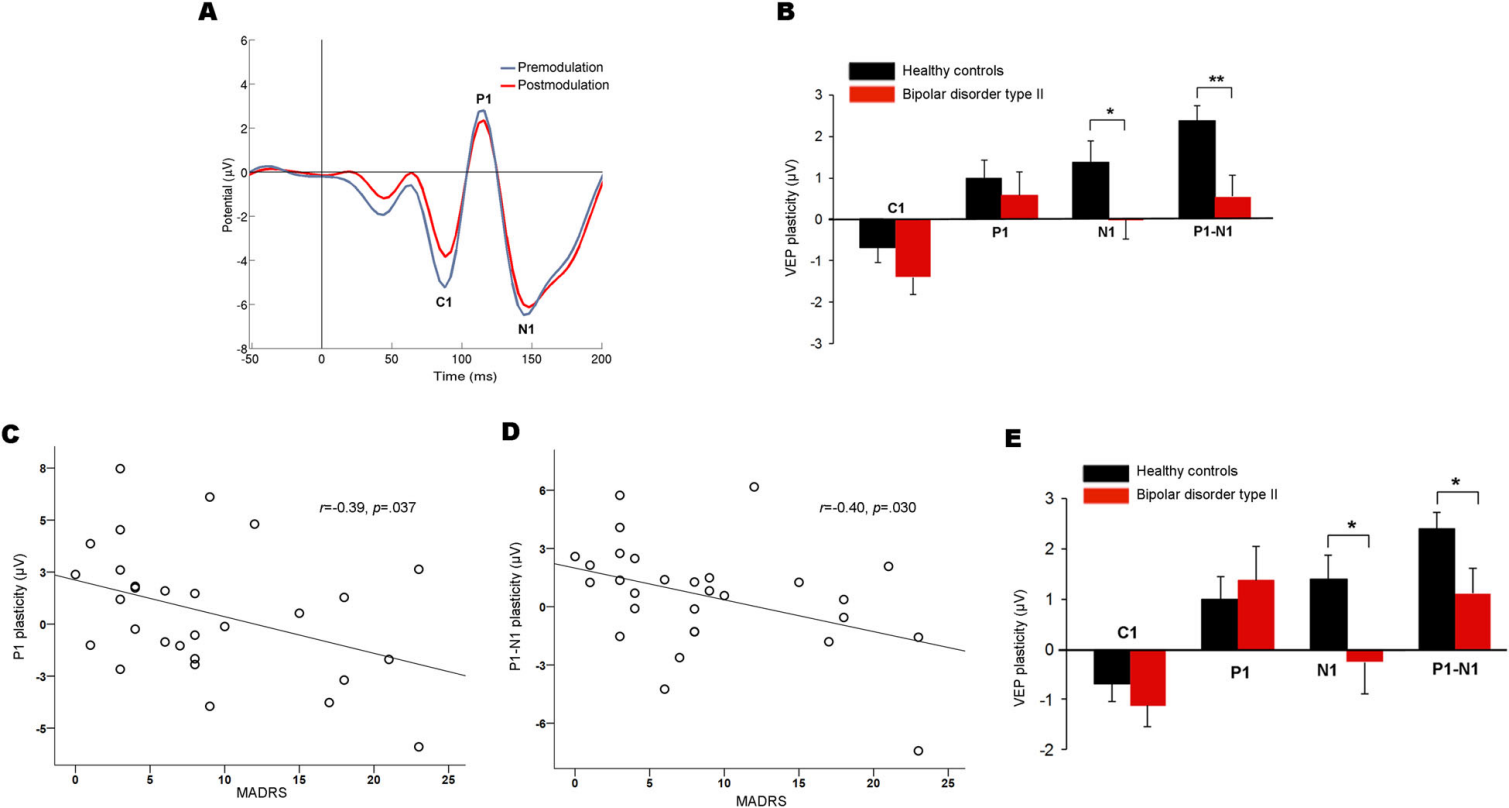
VEP plasticity of the cross-sectional sample at follow-up. **a** Grand average premodulation (blue) and postmodulation (red) VEP in patients with BD type II (*n* = 29). In contrast to controls, there was no significant P1, N1, or P1–N1 plasticity in the patient group. The VEPs for the controls are shown in Fig. 2a (the longitudinal and cross-sectional sample at follow-up was identical. **b** N1 and P1–N1 plasticity were significantly reduced in patients with BD type II relative to controls. P1–N1 plasticity remained reduced in patients after controlling for saliva cortisol and premodulation amplitudes. *p = 0.04 **p = 0.005. Error bars represent the s.e.m. **c** There were significant negative correlations between P1 plasticity and MADRS score and **d** P1–N1 plasticity and MADRS score in patients with BD type II, indicating stronger impairments in plasticity in more severely depressed patients. **e** N1 and P1–N1 plasticity were significantly reduced in euthymic patients with BD type II (*n* = 19) relative to controls; these reductions remained significant after controlling for saliva cortisol and premodulation amplitudes. *p < 0.05. Error bars represent the s.e.m. VEP, visual evoked potential. BD, bipolar disorder. MADRS, Montgomery–Asberg Depression Rating Scale

In the cross-sectional sample at follow-up, controls (Fig. 2a) and patients (Fig. 4a) showed the expected C1 at 87.4 ± .7 msec, P1 at 114.2 ± 0.6 msec, and N1 at 149.8 ± 1.5 msec after the checkerboard reversal. Patients had significantly greater N1 (*F*_1,56_ = 4.33, *p* = 0.04) and P1–N1 (*F*_1,56_ = 13.74, *p* < 0.001) amplitudes at the premodulation blocks than controls. There were no significant differences in premodulation N1 (13.6 μV and 9.9 μV in unmedicated and medicated patients, respectively) or P1–N1 (16.7 μV and 14.5 μV in unmedicated and medicated patients) amplitudes between unmedicated and medicated patients (see also Tables S1 and S2 for details). Premodulation N1 amplitude was larger in unmedicated patients than in controls and there were no significant difference in N1 amplitude when medicated patients and controls were compared (Table S1). Premodulation P1–N1 amplitude was significantly increased in both unmedicated and medicated patients relative to controls (both *p* < 0.05; Table S2). No significant group differences were found for the C1 or P1 premodulation amplitudes (both *p* > 0.05). Patients had lower C1 amplitudes at the postmodulation blocks than premodulation (*F*_1,26_ = 11.73, *p* = 0.002); no P1, N1, or P1–N1 plasticity was found in the patient group (all *p* > 0.32); however, there was a significant effect of modulation on P1–N1 amplitude in the first postmodulation block (*F*_1,28_ = 10.74, *p* = 0.003; Figure S2B).There was a significant correlation between modulation of P1 and N1 amplitudes in patients (*r* = −0.53, *p* = 0.003). There was significantly reduced plasticity of the N1 (*F*_1,56_ = 4.26; *p* *=* 0.04) and the P1–N1 amplitudes (*F*_1,56_ = 8.76; *p* *=* 0.005) in patients relative to controls (Fig. 4b). For P1–N1 plasticity, the group difference was significant for the first (*F*_1,56_ = 5.24, *p* = 0.026), second (*F*_1,56_ = 5.42, *p* = 0.024) and last postmodulation block (*F*_1,56_ = 14.36, *p* < 0.001, surviving Bonferroni correction for the six blocks tested). There was no significant group-salivary cortisol level interaction or group-premodulation amplitude interaction for P1–N1 plasticity (both *p’s* > 0.3), and the group difference remained significant after adjusting for these covariates (*F*_1,51_ = 9.16, *p* = 0.004), whereas the N1 plasticity did not (*p* = 0.10). There was no significant effect of medication use (controls vs. unmedicated vs. medicated patients) on N1 plasticity (*F*_2,55_ = 2.84, *p* = 0.067), however, there was a significant main effect of medication on P1–N1 plasticity (*F*_2,55_ = 4.30, *p* = 0.018). Post hoc analyses demonstrated significantly reduced plasticity in medicated patients (*n* = 22) relative to controls (*p* = 0.027, Bonferroni corrected). There was, however, no significant difference between unmedicated patients (*n* = 7) and control subjects (*p* = 0.199, Bonferroni corrected) or between medicated and unmedicated patients (*p* = 1.00, Bonferroni corrected; see also Tables S3 and S4 for details). Then, we found negative associations between P1 and P1–N1 plasticity and MADRS score in patients, indicating stronger impairments in plasticity in more severely depressed patients (*r* = −0.39, *p* = 0.04 and *r* = −0.40, *p* = 0.03; Fig. 4c, d). There were no significant associations between VEP plasticity and YMRS score in patients (all *p* > 0.05; Figure S3). Next, we found decreased N1 (*F*_1,46_ = 4.51, *p* *=* 0.04) and P1–N1 (*F*_1,46_ = 4.83, *p* *=* 0.03) plasticity in the euthymic patients (*n* = 19) relative to controls (Fig. 4e), which remained significantly reduced after adjusting for saliva cortisol and premodulation amplitudes (all *p* < 0.05). There were no significant differences in VEP plasticity between patients with and without panic disorder or social phobia (all *p* > 0.05).

#### VEP plasticity and saliva cortisol

In controls, there was a positive association between averaged saliva cortisol and P1–N1 plasticity (*r* = 0.43, *p* = 0.023; Fig. 3b) and a positive association between the cortisol awakening response and P1–N1 plasticity (*r* = 0.44, *p* = 0.018; Fig. 3c), indicating greater plasticity with increasing cortisol levels. In patients, there was a trending positive correlation between averaged saliva cortisol and P1–N1 plasticity that did not reach statistical significance (*rho* = 0.33, *p* = 0.096).

## Discussion

Plasticity of the VEP is a promising assay for noninvasive examination of cortical LTP-like synaptic processes^24,25^. In the present study of VEP plasticity in individuals with BD type II and controls, there were three main findings. First, we reproduced impaired VEP plasticity in BD type II at the follow-up examinations. We also found that VEP plasticity was reduced in euthymic patients and was negatively correlated with depression severity. Second, we showed that saliva cortisol was increased in BD type II, that VEP plasticity remained impaired in patients after controlling for saliva cortisol, and that saliva cortisol was positively correlated with plasticity of the VEP in controls. Finally, plasticity of the P1 and N1 components of the VEP, but not the P1–N1 component, exhibited moderate temporal stability in healthy individuals when baseline and follow-up examinations were compared.

Although its precise neural basis remains unknown, BD has been conceptualized as a genetically influenced disorder of synaptic function and plasticity in limbic-cortical neural networks involving the amygdala, hippocampus, and prefrontal cortices^9,14,45^. In support of this hypothesis, genetic studies have linked BD risk to genes implicated in synaptic function and plasticity regulation^46–51^. Further evidence for impaired synaptic function and plasticity in BD was found in rodent models of depression (which may reflect mechanisms relevant for the depressive phases of BD)^16,17^, in studies of the cellular targets of antidepressants and mood stabilizers^18,22,52,53^, and in post mortem studies of BD^54,55^.

Consistent with impaired synaptic function in BD, we previously observed decreased VEP plasticy in BD type II at the baseline examination^29^. Specifically, we found plasticity of the P1, the N1, and the P1–N1 amplitudes in controls and reduced P1–N1 plasticity in patients^29^. In the present study, we reproduced impaired P1–N1 plasticity in an overlapping sample of individuals with BD type II at follow-up on average 2.2 years after the baseline examinations^29^. We also found that VEP plasticity was decreased in euthymic patients. Moreover, P1 and P1–N1 plasticity correlated negatively with MADRS score, indicating lower plasticity in more severely depressed patients. Together, these results suggest that VEP plasticity is impaired in the euthmic phases of BD type II and may further deteriorate during depressive episodes. In contrast, there were no significant associations between hypomania severity and VEP plasticity. Yet, the hypomania symptoms of the patients were generally mild and further studies are needed to clarify the relationship between hypomania and VEP plasticity.

Another finding of the present study was increased saliva cortisol in BD type II. This observation is consistent with two recent meta-analysis, which found elevated saliva and blood cortisol in BD, particularly in euthymic and manic patients^56,57^. These studies involved individuals with BD type I or mixed samples of patients with BD type I or II^56,57^ and we are not aware of any previous cortisol study that has been limited to BD type II.

The effects of glucocorticoids on synaptic function and plasticity oftentimes follow an inverted U-shaped curve^37,38^. At low and high levels, glucocorticoids can impair synaptic function and plasticity, whereas normal glucocorticoid concentrations facilitate synaptic plasticity processes, such as LTP. These synaptic corticosteroid effects are likely mediated by both non-genomic, e.g., by pre- and postsynaptic modulation of glutamatergic transmission, and genomic mechanisms^37,38^. The well-established effects of glucocorticoids on synaptic function raise the possibility that the elevated cortisol underlies VEP plasticity impairments in BD type II. However, we found no significant association between saliva cortisol and VEP plasticity in the individuals with BD type II and their plasticity reduction remained significant after controlling group analyses for cortisol. Together, these findings indicate that impaired plasticity of the VEP in BD type II is not caused by elevated cortisol and other potential mechanisms should be addressed in future studies. In particular, BD risk genes have been linked to synaptic function and plasticity regulation^46–51^ and investigations of whether and how BD risk variants affect plasticity of the VEP are warranted.

We also found significant correlations between VEP plasticity and averaged saliva cortisol and the cortisol awakening response in controls (Fig. 3). Although speculative, these positive associations could reflect the ascending part of an inverted U-shaped relationship between cortisol and synaptic plasticity. This hypothesis could be tested in future studies of VEP plasticity by including more individuals with higher stress and cortisol levels than the present work. To our knowledge, there is no other study of cortisol and plasticity of the VEP, yet two previous studies reported significant relationships between cortisol and TMS-induced motor cortex plasticity^44,58^. Sale et al.^58^ found that motor cortex plasticity was greater in the evening (when endogenous cortisol is lower) than in the morning and that an oral dose of hydrocortisone blocked the motor cortex plasticity. Clow et al.^44^ observed a positive association between the cortisol awakening response and TMS-induced motor cortex plasticity, consistent with the results of the present study.

Based on the current understanding of mood regulation in humans^59,60^, it is unlikely that impaired visual cortex synaptic plasticity is a central pathophysiological mechanism in BD type II. Two important, yet unresolved questions are therefore (1) to what extent does VEP plasticity reflect plasticity in brain regions believed to be important in mood disorders, such as prefrontal and temporal cortices and (2) are the putative cortical synaptic impairments in bipolar disorders confined to specific mood regulation-related regions or widespread? To our knowledge, no study has examined the association between synaptic plasticity in prefrontal and temporal regions and VEP plasticity. However, one recent investigation found significant association between motor cortex plasticity and VEP plasticity^61^. Moreover, previous post mortem studies found evidence for synaptic impairments in bipolar disorders in several cortical regions, including prefrontal, temporal, and visual cortices^55,62–66^. In addition, the significant association between depression severity and visual plasticity found in the present study supports the notion that VEP plasticity might be used as an indirect measure of synaptic impairments in mood regulation-related cortical regions. Nevertheless, more research is needed to clarify the relationship between VEP plasticity and synaptic plasticity in cortices implicated in mood regulation.

An unexpected finding of the present study was that patients had greater N1 and P1–N1 premodulation amplitudes than controls at follow-up. We examined whether use of psychotropic drugs could underlie the premodulation amplitude increases and found, if anything, a trend toward smaller premodulation amplitudes in medicated than in unmedicated patients (see also Tables S1 and S2 for details). In contrast, there were no group differences in the premodulation amplitudes at baseline^29^. The premodulation amplitude increase in patients at follow-up should therefore be considered cautiously and need to be confirmed by future research. Another important question is whether the larger premodulation amplitudes in patients at follow-up could be related to their VEP plasticity reduction, e.g., owing to a ceiling effect. However, the P1–N1 plasticity remained significantly reduced in patients after adjusting for premodulation amplitude. In addition, P1–N1 plasticity was also significantly reduced in patients at baseline when there were no group differences in the premodulation amplitudes. Altogether, it seems unlikely that the increased premodulation amplitude in patients underlie their impaired P1–N1 plasticity observed at follow-up.

The field of noninvasive LTP-like cortical plasticity assessment in humans is young and mainly encompasses repetitive visual or auditory stimulation-induced plasticity in sensory cortices (e.g., VEP plasticity), motor cortex plasticity induced by TMS or transcranial direct current stimulation (tDCS), and sleep slow wave activity (SWA) of the EEG^30,36,67–76^. Previous studies found that TMS-induced motor cortex plasticity was decreased in MDD^77^ and that tDCS might increase motor cortex plasticity in depressed individuals^78^. Sleep SWA is another potential EEG-based index of cortical synaptic plasticity^72–76^ and increased SWA during sleep has been linked to the rapid antidepressant response to ketamine treatment in MDD^79^. Moreover, recent studies indicate that TMS and tDCS can alter SWA^80,81^ and that acoustic stimulation might enhance SWA during sleep^74^. Each of these methodologies has strengths and limitations and we chose VEP plasticity in our studies because of its feasibility (e.g., no requirement of sleep or magnetic stimulation) and since detailed studies found that VEP plasticity in rodents exhibits core features of LTP^31–33^.

The temporal stability of these noninvasive plasticity indices remains to be clarified and there has, to our knowledge, been no previous longitudinal study of sensory cortex LTP-like plasticity. There is also a scarcity of studies examining the test–retest reliability of other noninvasive plasticity indices and the limited TMS-induced motor cortex plasticity literature observed substantial variability^82^. For example, Fratello et al.^83^ found low temporal stability when TMS-induced motor cortex plasticity was measured twice in healthy volunteers with a 1 week test–retest interval (*ICC* = 0.05). Thus, the results of the present study indicating moderate temporal stability of both the P1 and the N1 plasticity (*ICC* between 0.5 and 0.6) in controls with a test–retest interval of 2.2 years period are promising. These *ICC*’s are also comparable to the reliability commonly found in functional magnetic resonance imaging studies with test–retest intervals of weeks to months (*ICC* usually between 0.33 and 0.66)^84^. We note, however, that the temporal stability of P1–N1 was low in the controls of the current study. This finding could be due to P1 and N1 modulation representing two independent plasticity indices. However, we found that P1 and N1 plasticity were significantly negatively correlated, which suggests that they may reflect at least partly overlapping mechanisms. We therefore speculate that the poor reliability might be related to the fact that P1–N1 includes the variability of both the P1 and the N1 component; this composite measure might thus have lower temporal stability than the individual VEP components. Thus, although the present results are promising, more work is needed to clarify the test–retest reliability of VEP plasticity in humans.

The present study comes with several limitations. First, the sample size was modest and larger studies are needed to clarify the relationships between VEP plasticity and comorbid psychiatric illnesses and illness course. Second, there were no significant differences in VEP plasticity between medicated and unmedicated patients, yet the number of unmedicated patients was small (*n* = 7), at follow-up. Further studies are therefore needed to fully clarify the effects of psychotropic drugs on VEP amplitudes and plasticity. Third, the present study did not include a BD type I or an MDD comparison group. More research is therefore required to assess whether the VEP plasticity impairments observed in patients of the present study are specific for BD type II or common neurobiological characteristics of mood disorders. Fourth, the precise neural mechanisms underlying VEP plasticity in humans remain to be fully clarified. However, rodent and human studies strongly suggest that plasticity of the VEP reflect cortical processes closely related to LTP^24,25,31–33^. Fifth, our plasticity paradigm did not include a control stimulus to test whether VEP modulation was dependent on stimulation properties. However, frequency- and pattern-specific VEP potentiation has been demonstrated previously for the current paradigm^28^. Sixth, we employed a limited number of electrodes. The use of high-density EEG or magnetoencephalography may increase our understanding of how modulation of cortical excitability might change neural circuitry in mood disorders. Seventh, whereas VEP plasticity was examined longitudinally, saliva cortisol was measured only at follow-up. Finally, future research could also examine whether other stress-related indices are associated with VEP plasticity, such as hair cortisol, heart rate variability, and alpha-amylase levels.

In conclusion, the present study provides additional evidence for impaired LTP-like cortical plasticity in BD type II, suggests that impaired cortical plasticity is present in the euthymic phases of the illness and may further deteriorate during depressive episodes, and indicates that elevated cortisol does not underlie the plasticity impairment. The results also suggest a positive association between the VEP plasticity and saliva cortisol in controls, possibly reflecting an inverted U-shaped relationship between cortisol and synaptic plasticity. From a methodological perspective, converging lines of evidence indicate that synaptic dysfunction is a central pathophysiological mechanism across psychiatric illnesses^9–13^ and there is therefore a substantial need for techniques, which enable assessment of cortical synaptic function and plasticity in humans. The previous^24,25,28–30^ and present works together suggest that VEP plasticity is an accessible method for noninvasive studies of LTP-like cortical processes, which may help elucidate the pathophysiological and clinical significance of synaptic dysfunction in psychiatric disorders.

## Electronic supplementary material

Supplement
