# Supplementary material for: Longitudinal and cross-sectional investigations of long-term potentiation-like cortical plasticity in bipolar disorder type II and healthy individuals
Source: Transl Psychiatry. 2018 May 24;8:103. doi: 10.1038/s41398-018-0151-5 (PMC5966393; doi:10.1038/s41398-018-0151-5)
Supplement: Supplementary file 1 — Supplement [file 41398_2018_151_MOESM1_ESM.docx]

Longitudinal and Cross-Sectional Investigations of Long-Term Potentiation-Like Cortical Plasticity in Bipolar Disorder Type II and Healthy Individuals

**SUPPLEMENT**

**SUPPLEMENTARY FIGURES**

**Figure S1.** VEP plasticity of the longitudinal sample at baseline. (**A**) Grand average premodulation (blue) and postmodulation (red) VEP in controls (*n*=29). There was significant P1, N1 and P1-N1 plasticity. (**B**) Grand average premodulation (blue) and postmodulation (red) VEP in patients with BD type II (*n*=16). In contrast to controls, there was no P1, N1, or P1-N1 plasticity in patients (all *p*>.05). (**C**) VEP plasticity in patients with BD type II and controls. P1-N1 plasticity was significantly reduced in patients relative to controls. ***p*=.001. Error bars represent the s.e.m. Note that the grand average ERP displayed in A and B represents mean amplitude across participants for each (absolute) time-point following stimulus onset, while all statistical analyses were conducted on peak amplitudes individually determined for each participants (displayed in C). Also, two patients included in B did not display a reliable C1 component, and was hence excluded from the C1 analysis in C. Thus, the values in A and B do not strictly correspond to those in C, especially for the C1 component. VEP, visual evoked potential. BD, bipolar disorder.


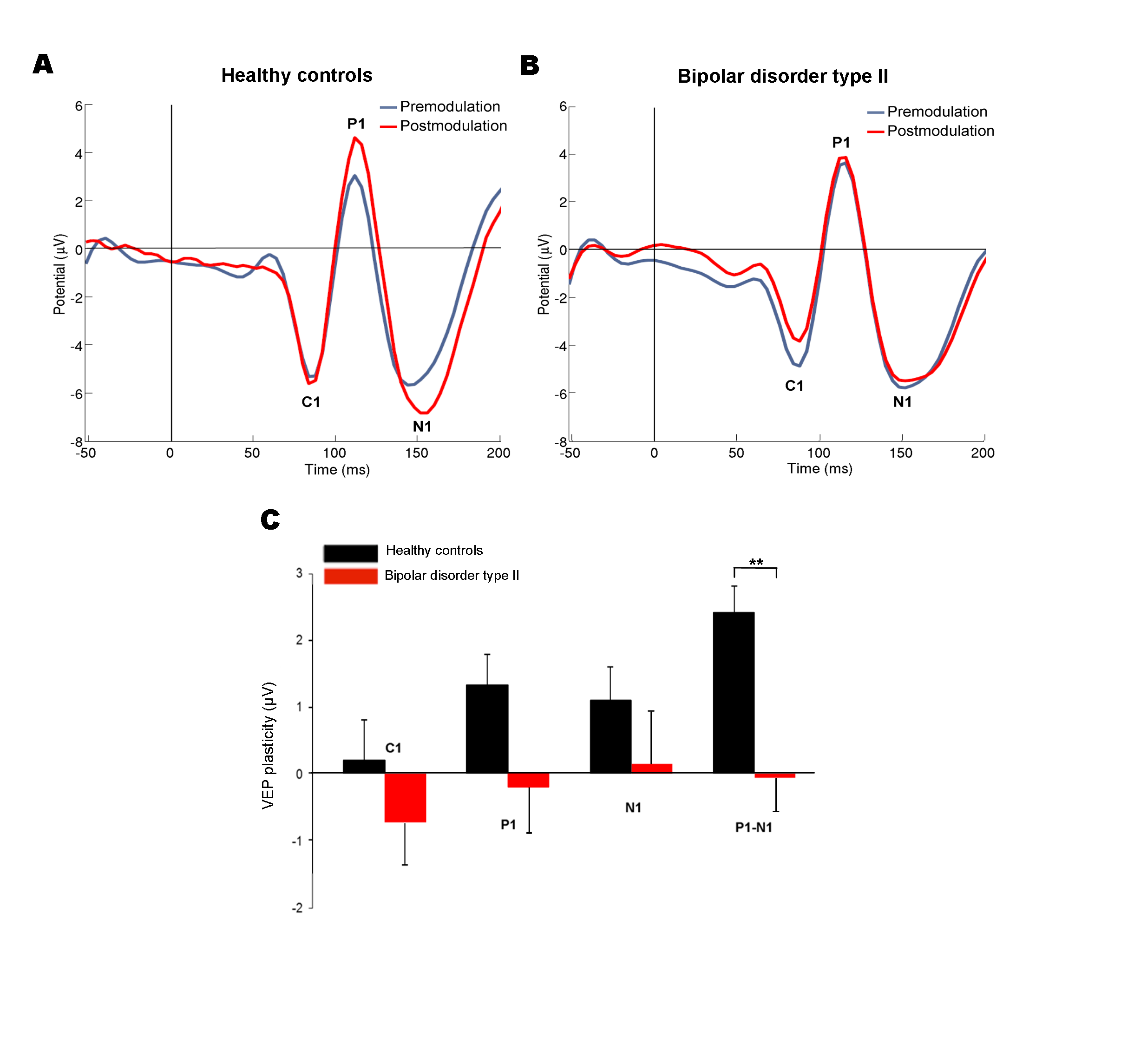


**Figure S2.** Change in P1-N1 amplitude at the six postmodulation blocks relative to premodulation.**(A)** The postmodulation P1-N1 amplitudes differed significantly from the premodulation amplitude for all six postmodulation blocks (five out of six surviving Bonferroni correction) in healthy individuals. * *p*=.32 ** *p*=.001 *** *p*<.001 **(B)** Only the P1-N1 amplitude at the first postmodulation block differed significantly from the premodulation amplitude in bipolar disorder type II. * *p*=.003

**
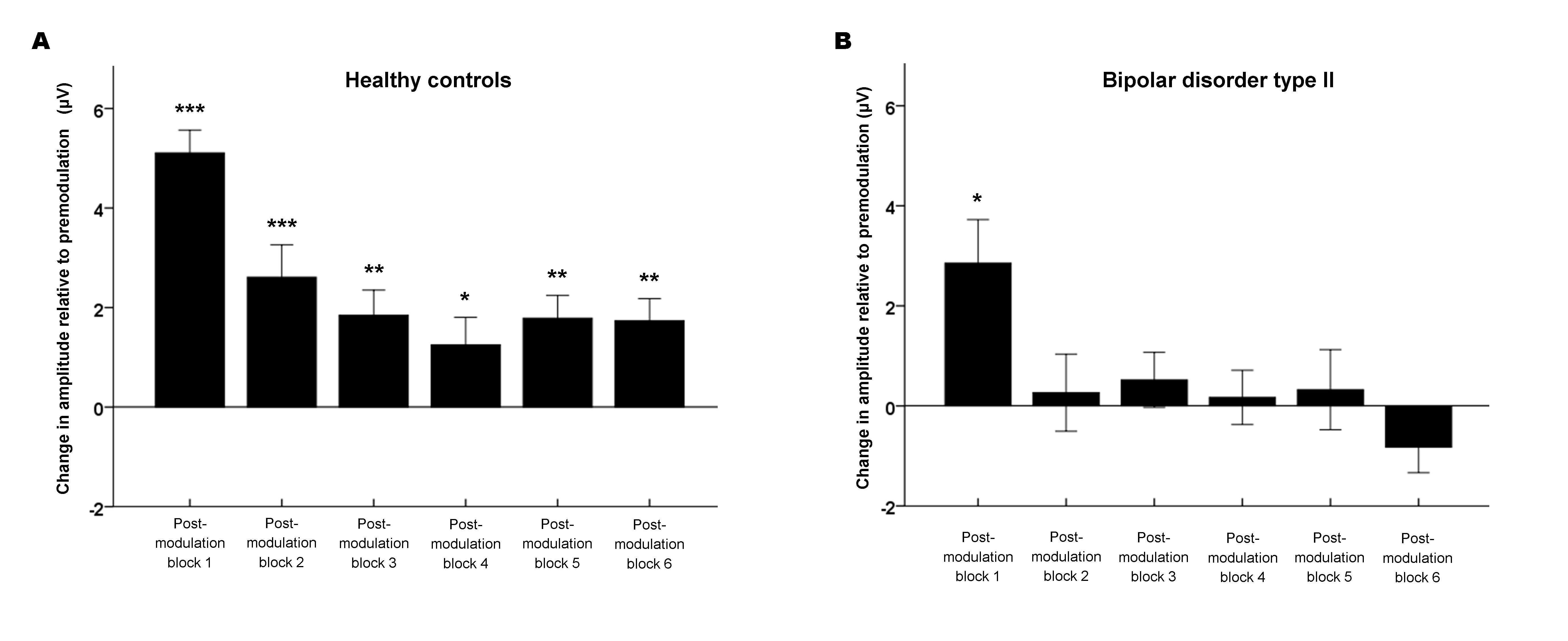
**

**Figure S3.** VEP plasticity of the cross-sectional sample at follow-up. (**A**) There were no correlations between YMRS score and P1 plasticity or (**B**) P1-N1 plasticity in patients with bipolar disorder type II. VEP, visual evoked potential. YMRS, Young Mania Rating Scale.


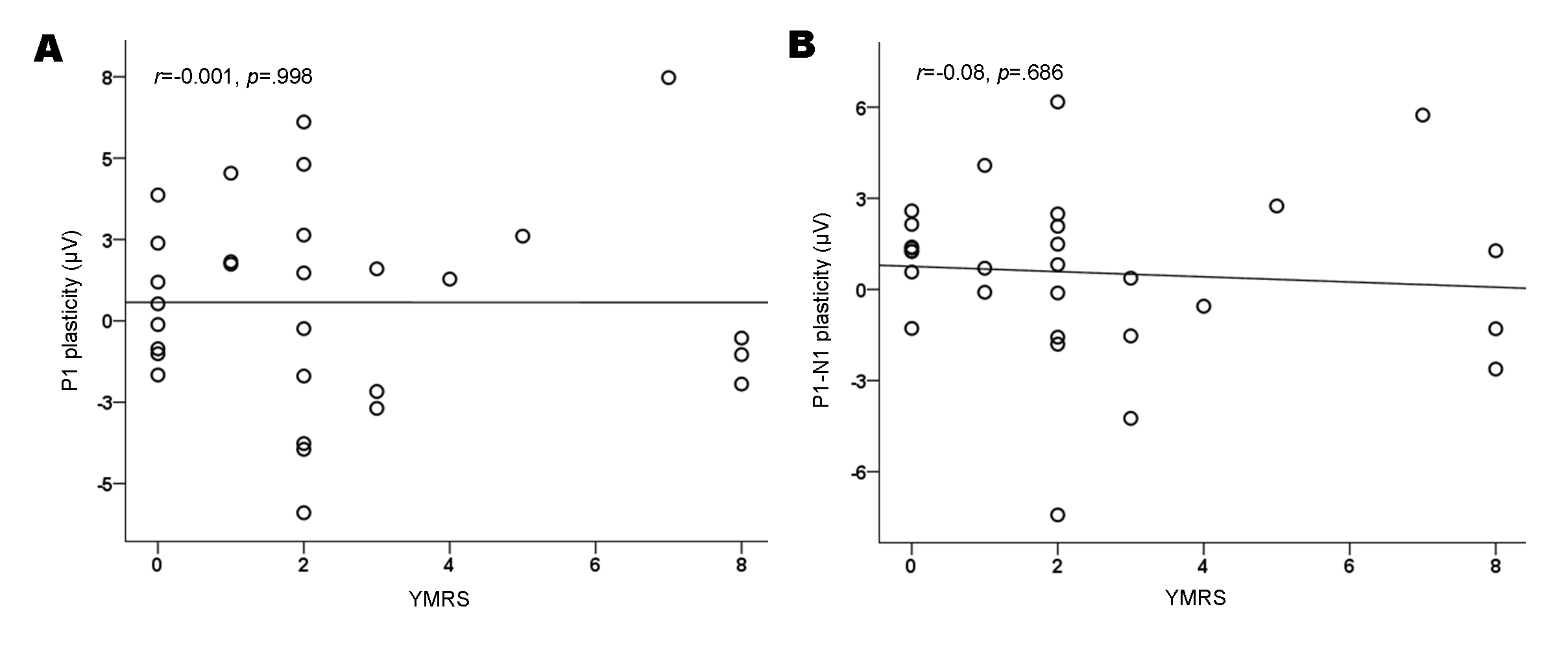


**SUPPLEMENTARY TABLES**

**Table S1**. Effects of medication use on premodulation N1 amplitude in patients of the cross-sectional sample at follow-up. Only psychotropic drugs with current users of n ≥ 3 of the sample are shown.

| **Medication** | ***n* (%)** | **Amplitude (μV (SD)) in patients not using drug (NU)** | **Amplitude (μV (SD)) in patients using drug (U)** | **Mean amplitude (μV (SD)) in controls (C)** | **Statistics** | **Post hoc test, *p*-value (Bonferroni corrected)** |
| --- | --- | --- | --- | --- | --- | --- |
| **All antidepressants** | 7 (24.1) | -11.8 (6.8) | -7.8 (4.3) | -7.9 (4.0) | *F*_2,55_=3.72  *p*=.031 | NU>C, .036 |
| **SSRIs** | 3 (10.3) | -11.6 (6.3) | -3.9 (2.4) | -7.9 (4.0) | *F*_2,55_=5.41  *p*=.007 | NU>U, .050 NU>C, .028 |
| **Lamotrigine** | 16 (55.2) | -12.8 (7.6) | -9.2 (4.9) | -7.9 (4.0) | *F*_2,55_=4.02  *p*=.024 | NU>C, .019 |
| **Quetiapine** | 3 (10.3) | -10.6 (6.6) | -12.3 (5.0) | -7.9 (4.0) | *F*_2,55_=2.26  *p*=.114 | n.a. |
| **Any medication** | 22 (75.9) | -13.6 (10.0) | -9.9 (2.4) | -7.9 (4.0) | *F*_2,55_=3.58  *p*=.035 | NU>C, .036 |

SD, standard deviation

**Table S2.**  Effects of medication use on premodulation P1-N1 amplitude in patients of the cross-sectional sample at follow-up. Only psychotropic drugs with current users of n ≥ 3 of the sample are shown.

| **Medication** | **n (%)** | **Amplitude (μV (SD)) in patients not using drug (NU)** | **Amplitude (μV (SD)) in patients using drug (U)** | **Amplitude (μV (SD)) in controls (C)** | **Statistics** | **Post hoc test, *p*-value (Bonferroni corrected)** |
| --- | --- | --- | --- | --- | --- | --- |
| **All antidepressants** | 7 (24.1) | 15.5 (7.0) | 13.5 (6.6) | 9.3 (4.7) | *F*_2,55_=7.15  *p*=.002 | NU>C, .001 |
| **SSRIs** | 3 (10.3) | 15.4 (7.1) | 11.4 (2.0) | 9.3 (4.7) | *F*_2,55_=7.53  *p*=.001 | NU>C, .001 |
| **Lamotrigine** | 16 (55.2) | 15.3 (8.1) | 14.8 (6.0) | 9.3 (4.7) | *F*_2,55_=6.77  *p*=.002 | NU>C, .012 U>C, .012 |
| **Quetiapine** | 3 (10.3) | 15.4 (7.1) | 12.1 (3.8) | 9.3 (4.7) | *F*_2,55_=7.27  *p*=.002 | NU>C, .001 |
| **Any medication** | 22 (75.9) | 16.7 (9.5) | 14.5 (6.0) | 9.3 (4.7) | *F*_2,55_=7.20  *p*=.002 | NU>C, .013  U>C, .009 |

SD, standard deviation

**Table S3.** Effects of medication use on N1 plasticity in patients of the cross-sectional sample at follow-up. Only psychotropic drugs with current users of n ≥ 3 of the sample are shown.

| **Medication** | ***n* (%)** | **Amplitude change (μV (SD)) in patients not using drug (NU)** | **Amplitude change (μV (SD)) in patients using drug (U)** | **Amplitude change (μV (SD)) in controls (C)** | **Statistics** | **Post hoc test, *p*-value (Bonferroni corrected)** |
| --- | --- | --- | --- | --- | --- | --- |
| **All antidepressants** | 7 (24.1) | -.24 (2.6) | .68 (2.1) | 1.39 (2.7) | *F*_2,55_=2.45  *p*=.096 | n.a. |
| **SSRIs** | 3 (10.3) | -.34 (2.5) | 2.82 (0.5) | 1.39 (2.7) | *F*_2,55_=4.37  *p*=.017 | NU<C, .041 |
| **Lamotrigine** | 16 (55.2) | .56 (2.3) | -.48 (2.7) | 1.39 (2.7) | *F*_2,55_=2.72  *p*=.075 | n.a. |
| **Quetiapine** | 3 (10.3) | -.14 (2.6) | 1.01 (1.1) | 1.39 (2.7) | *F*_2,55_=2.37  p=.103 | n.a. |
| **Any medication** | 22 (75.9) | 1.00 (2.8) | -.34 (2.4) | 1.39 (2.7) | *F*_2,55_=2.84  *p*=.067 | n.a. |

SD, standard deviation

**Table S4**. Effects of medication use on P1-N1 plasticity in patients of the cross-sectional sample at follow-up. Only psychotropic drugs with current users of n ≥ 3 of the sample are shown.

| **Medication** | ***n* (%)** | **Amplitude change (μV (SD)) in patients not using drug (NU)** | **Amplitude change (μV (SD)) in patients using drug (U)** | **Amplitude change (μV (SD)) in controls (C)** | **Statistics** | **Post hoc test, *p*-value (Bonferroni corrected)** |
| --- | --- | --- | --- | --- | --- | --- |
| **All antidepressants** | 7 (24.1) | .64 (2.5) | .26 (3.7) | 2.39 (1.9) | *F*_2,55_=4.38  *p*=.017 | NU<C, .037 |
| **SSRIs** | 3 (10.3) | .47 (2.9) | 1.30 (0.9) | 2.39 (1.9) | *F*_2,55_=4.49  *p*=.016 | NU<C, .012 |
| **Lamotrigine** | 16 (55.2) | .93 (2.0) | .24 (3.3) | 2.39 (1.9) | *F*_2,55_=4.66  *p*=.014 | U<C, .016 |
| **Quetiapine** | 3 (10.3) | .51 (2.8) | .87 (2.2) | 2.39 (1.9) | *F*_2,55_=4.34  *p*=.018 | NU<C, .016 |
| **Any medication** | 22 (75.9) | .51 (1.5) | .57 (3.1) | 2.39 (1.9) | *F*_2,55_=4.30  *p*=.018 | U<C, .027 |

SD, standard deviation

**Table S5**. Effects of adding a psychotropic drug on VEP plasticity changes in patients from baseline to follow-up.

| **VEP component** | **Amplitude change from baseline to follow-up (μV (SD)) in patients who added medication (*n*=5)** | **Amplitude change from baseline to follow-up (μV (SD)) in other patients (*n*=11)** | **Amplitude change from baseline to follow-up (μV (SD)) in controls (*n*=29)** | **Statistics** |
| --- | --- | --- | --- | --- |
| **C1** | -1.82 (3.7) | .83 (1.7) | -.97 (3.2) | *F*_2,42_=.78  *p*=.465 |
| **P1** | -.54 (5.7) | 1.10 (3.1) | .32 (2.4) | *F*_2,42_=.43  p=.657 |
| **N1** | -1.47 (4.9) | 1.61 (2.5) | .30 (2.4) | *F*_2,42_=1.98  p=.151 |
| **P1N1** | .93 (2.4) | -.51 (1.3) | .02 (2.8) | *F*_2,42_=.70  *p*=.503 |

SD, standard deviation
